# Morning SARS-CoV-2 Testing Yields Better Detection of Infection Due to Higher Viral Loads in Saliva and Nasal Swabs upon Waking

**DOI:** 10.1128/spectrum.03873-22

**Published:** 2022-10-26

**Authors:** Alexander Viloria Winnett, Michael K. Porter, Anna E. Romano, Emily S. Savela, Reid Akana, Natasha Shelby, Jessica A. Reyes, Noah W. Schlenker, Matthew M. Cooper, Alyssa M. Carter, Jenny Ji, Jacob T. Barlow, Colten Tognazzini, Matthew Feaster, Ying-Ying Goh, Rustem F. Ismagilov

**Affiliations:** a California Institute of Technology, Pasadena, California, USA; b City of Pasadena Public Health Department, Pasadena, California, USA; University of Mississippi Medical Center

**Keywords:** COVID-19, pandemic, diagnostic, screening, analytical sensitivity, outpatient, ambulatory, circadian rhythm, specimen collection, viral load, sample collection

## Abstract

Optimizing specimen collection methods to achieve the most reliable SARS-CoV-2 detection for a given diagnostic sensitivity would improve testing and minimize COVID-19 outbreaks. From September 2020 to April 2021, we performed a household-transmission study in which participants self-collected specimens every morning and evening throughout acute SARS-CoV-2 infection. Seventy mildly symptomatic participants collected saliva, and of those, 29 also collected nasal swab specimens. Viral load was quantified in 1,194 saliva and 661 nasal swab specimens using a high-analytical-sensitivity reverse transcription-quantitative PCR (RT-qPCR) assay. Viral loads in both saliva and nasal swab specimens were significantly higher in morning-collected specimens than in evening-collected specimens after symptom onset. This aspect of the biology of SARS-CoV-2 infection has implications for diagnostic testing. We infer that morning collection would have resulted in significantly improved detection and that this advantage would be most pronounced for tests with low to moderate analytical sensitivity. Collecting specimens for COVID-19 testing in the morning offers a simple and low-cost improvement to clinical diagnostic sensitivity of low- to moderate-analytical-sensitivity tests.

**IMPORTANCE** Our findings suggest that collecting saliva and nasal swab specimens in the morning immediately after waking yields higher SARS-CoV-2 viral loads than collection later in the day. The higher viral loads from morning specimen collection are predicted to significantly improve detection of SARS-CoV-2 in symptomatic individuals, particularly when using moderate- to low-analytical-sensitivity COVID-19 diagnostic tests, such as rapid antigen tests.

## INTRODUCTION

Although vaccination has substantially reduced hospitalizations and death from COVID-19, limited vaccine uptake and availability and the potential for breakthrough infections (particularly with novel viral variants) support the continued necessity for diagnostic testing and subsequent isolation of infected individuals (1, 2). Optimizing how diagnostics are used can enhance our ability to combat the COVID-19 pandemic.

Nasopharyngeal swab, anterior nares swab, midturbinate swab, oropharyngeal swab, buccal swab, gingival crevicular fluid, sputum, tracheal aspirate, and saliva have all been utilized and compared as diagnostic specimens for the detection of SARS-CoV-2 infection. Work done by many groups (3–5), including ours (6), has suggested that SARS-CoV-2 is detectable, albeit at low viral loads, in saliva before anterior nares nasal swab specimens. However, conflicting results have been reported in head-to-head comparisons of saliva to other specimen types in cross-sectional studies.

Lack of clarity on which specimen type is most reliable for SARS-CoV-2 detection is likely due to the dynamic nature of viral loads in different specimen types through the course of an infection (3, 6–11) and the differences in analytical sensitivity of diagnostic assays used in the comparisons. Currently available SARS-CoV-2 diagnostics span a wide (6 orders of magnitude) range of analytical sensitivities, from the reverse transcription-PCR (RT-PCR) PerkinElmer new coronavirus nucleic acid detection kit (limit of detection [LOD] of 180 nucleic acid amplification test detectable units [NDU]/mL) (12) to the Coris BioConcept rapid antigen lateral flow assay COVID-19 Ag Respi-Strip (LOD of ~4 × 10^7^ copies/mL) (13). Tests with relatively moderate analytical sensitivity (LOD of 10^4^ to 10^5^ copies/mL of specimen) or low analytical sensitivity (LOD of 10^5^ to 10^8^ copies/mL of specimen) are being increasingly used, particularly for at-home and rapid screening testing and in areas of the world with limited laboratory capacity (14–16).

How specimens are collected can also affect the detectability of SARS-CoV-2 in a specimen. Because SARS-CoV-2, like other pathogens, may exhibit circadian rhythms to replication kinetics (17, 18), we hypothesized that collection time may impact SARS-CoV-2 viral load in respiratory specimens and therefore detectability of infection. Simple, low-cost changes to specimen collection protocols that significantly improve the clinical sensitivity of COVID-19 diagnostics offer an immediately actionable opportunity to improve existing diagnostics, which would be particularly valuable in settings that rely on tests with low analytical sensitivity.

We conducted a COVID-19 household transmission study (9, 19) where participants prospectively self-collected saliva and nasal swab specimens twice per day (in the morning and in the evening). From mildly symptomatic participants, we compared SARS-CoV-2 viral loads in morning- and evening-collected specimens to determine if the time of day affected viral load, and if this could be leveraged to improve detection of SARS-CoV-2 infection.

## RESULTS

### Timing of morning and evening specimen collection.

**FIG 1:**
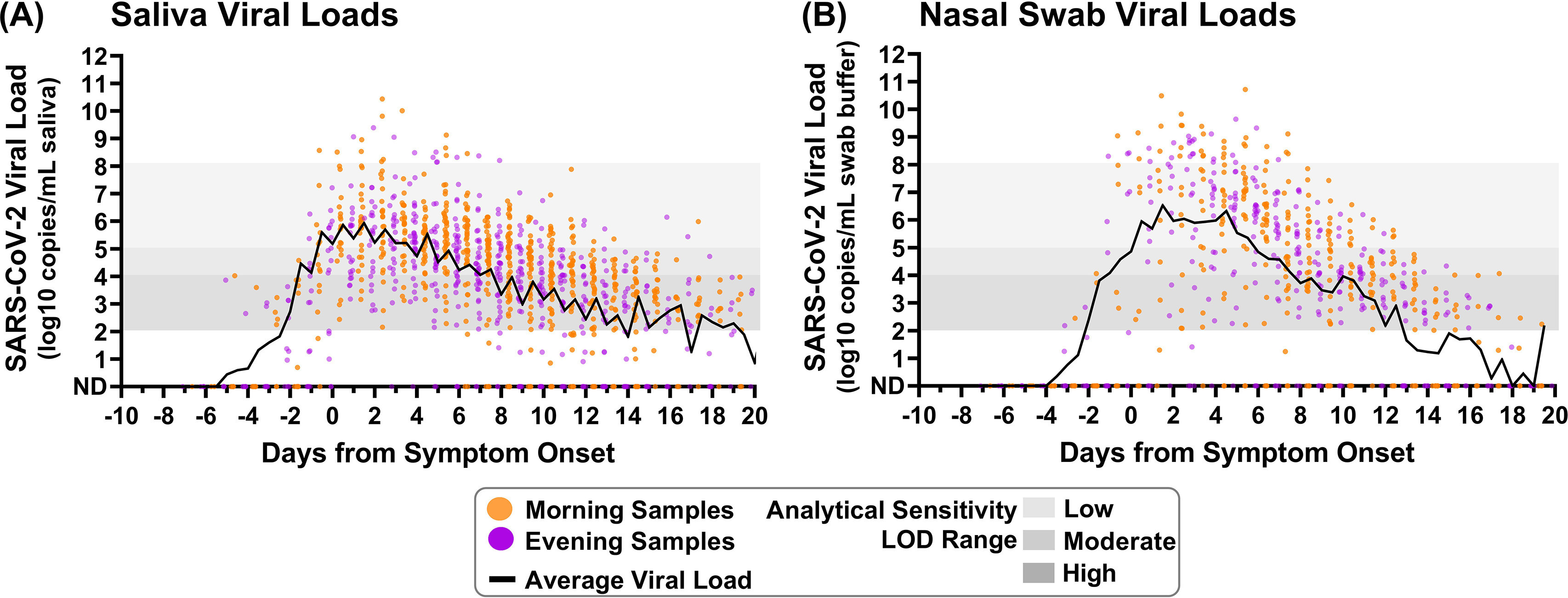
Saliva and nasal swab specimens collected in the morning and evening through the course of infection demonstrate differences in SARS-CoV-2 viral load. Black lines on each plot indicate the average viral load for each daily morning or evening specimen collection window. (A) Saliva specimen viral load (SARS-CoV-2 *N1* copies/milliliter of saliva) as measured by RT-qPCR is plotted relative to symptom onset for 1,194 specimens. (B) Nasal swab specimen viral load (*N1* copies/milliliter of swab buffer) as measured by RT-qPCR is plotted relative to symptom onset for 661 specimens. Specimens were designated morning (orange) if collected between 4 a.m. and 12 p.m. or evening (purple) if collected between 3 p.m. and 3 a.m. ND, not detected. Additional specimen details are provided in the supplemental material.

Viral load was quantified in 1,194 saliva specimens from 70 individuals and 661 nasal swab specimens from 29 individuals (Fig. 1). The distribution of collection times was roughly bimodal. Although each participant’s specimen collection time varied slightly throughout enrollment, nearly all (92%) participants had an average morning specimen collection time between 7 a.m. and 10 a.m. Evening collection time was more variable, but most participants (74%) had an average specimen collection time between 8 p.m. and 11 p.m. These patterns were used to delineate the morning and evening periods in the study: we defined sampling upon waking (4 a.m. to 12 p.m.) as morning and sampling before bed (3 p.m. to 3 a.m.) as evening (see Fig. S1 in the supplemental material).

### Saliva and nasal swab specimens exhibit higher viral loads in morning than evening collection across the course of acute, symptomatic illness.

Saliva and nasal swab viral load profiles from most individuals (Fig. S2 and S3) revealed a pattern of higher viral loads in specimens collected in the morning than in those collected in the evening. In specimens from some individuals (e.g., Fig. S2A and S3E), fluctuations in both SARS-CoV-2 and human *RNase P* markers were observed, whereas in others *RNase P* remained stable and SARS-CoV-2 viral load appeared to be independent of the host marker (e.g., Fig. S2AH and S3N).

Although direct comparison between all positive morning or evening specimens demonstrates greater target abundance for both SARS-CoV-2 *N1* (Fig. S4A and C) and human *RNase P* (Fig. S4B and D), this comparison would be skewed by participants who contributed more specimens and biased by sampling at different stages of the infection. To minimize these potential biases, the time of each specimen collection was aligned relative to the date of symptom onset for that participant before plotting both individual viral load datapoints (Fig. S2 and S3) and the average of log-transformed viral load values (Fig. 1A and B) for all saliva and nasal swab specimens in 12 hour time bins.

**FIG 2:**
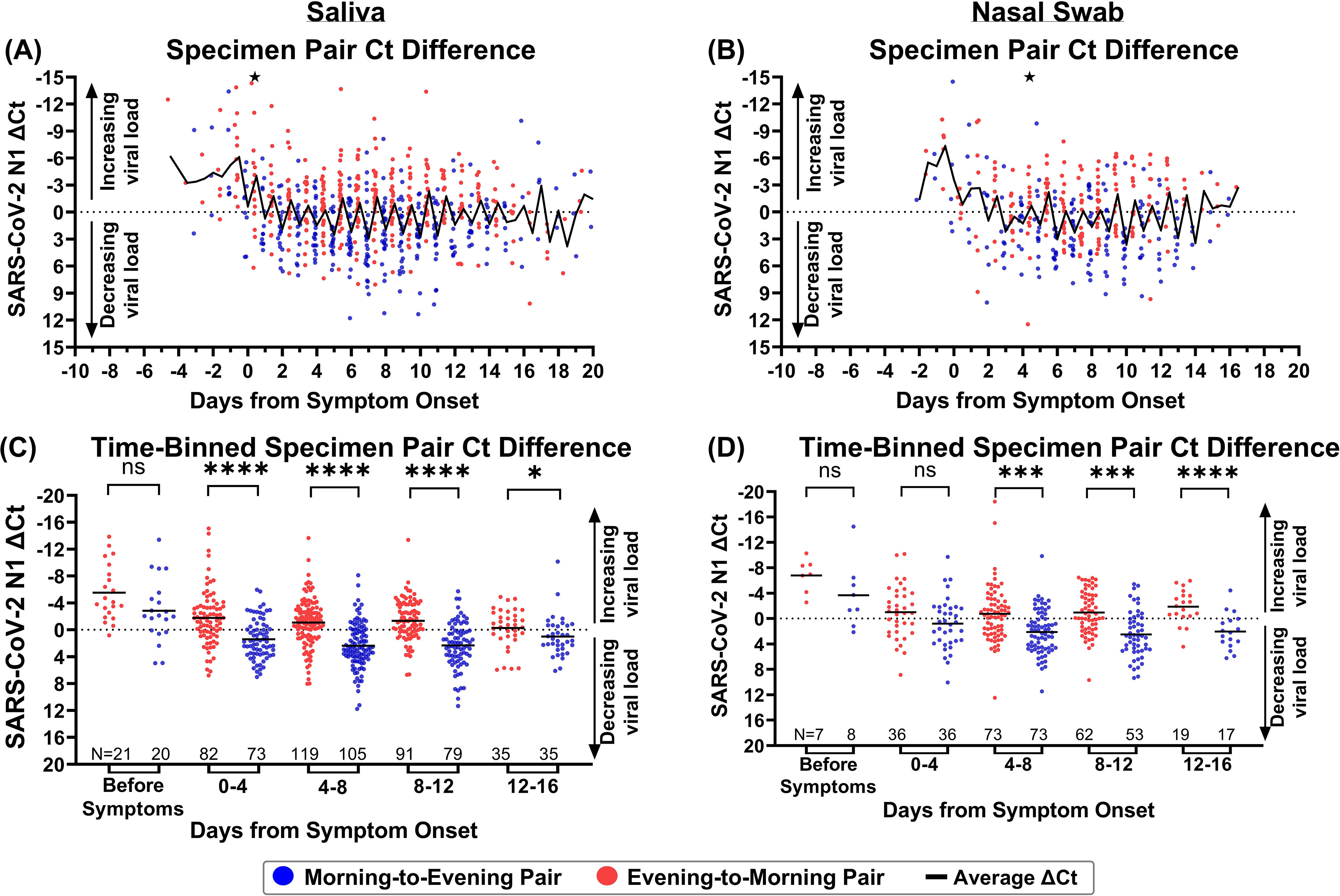
Morning viral loads are significantly higher than evening viral loads during most of SARS-CoV-2 infection. (A and B) The difference in *N1 C_T_* values (Δ*C_T_*) in 703 morning-to-evening and evening-to-morning successive saliva specimen pairs (A) and 365 morning-to-evening and evening-to-morning successive nasal swab specimen pairs (B), plotted relative to symptom onset. One point in panel A and one point in panel B had Δ*C_T_* values outside the *y* axis of the plot; these are represented as black stars at −15. (C and D) The difference in *N1 C_T_* values in 703 morning-to-evening and evening-to-morning sequential saliva (C) and nasal swab (D) specimen pairs relative to symptom onset. Morning-to-evening or evening-to-morning Δ*C_T_* values were then binned into presymptomatic or 4-day infection stages. The distributions of morning-to-evening and evening-to-morning Δ*C_T_* values for each infection stage bin were then statistically compared using the Wilcoxon matched-pair signed-rank test; ns, nonsignificant or insufficient data points to perform analysis; *, *P* < 0.05; **, *P* < 0.01; ****, *P* < 0.001. Black lines indicate average viral load. ND, not detected.

The averaged salivary viral load during each collection time point visually suggests higher viral loads in specimens collected in the morning than in the evening during both the presymptomatic and symptomatic phases of infection. This pattern was less apparent in the averaged nasal swab viral loads but can be seen when comparing the *N1* threshold cycle (*C_T_*) values between successive time points by calculating differences in *C_T_* (Fig. 2A and B). Only reverse transcription-quantitative PCR (RT-qPCR) *C_T_* values for pairs of successively collected morning-to-evening or evening-to-morning specimen were used to calculate the *C_T_* difference; negative or indeterminate specimens were included only if directly followed by a positive specimen collected in the presymptomatic phase of infection. A negative difference in *C_T_* values indicates that viral load was increasing relative to the previous measurement, whereas a positive difference indicates that viral load was decreasing relative to the previous measurement. Starting from symptom onset (day 0), saliva specimens collected in the morning typically exhibited a negative difference in *C_T_* values relative to their preceding evening specimens, whereas evening specimens consistently had a positive difference in *C_T_* values relative to their preceding morning specimens. This indicates that throughout the course of symptomatic infection, morning specimens typically result in relatively lower *C_T_* values (higher viral loads) than evening specimens.

To further illustrate the pattern observed in viral loads and changes in *C_T_* values, specimens were binned by infection stage: prior to symptom onset and in 4-day intervals relative to symptom onset. The 4-day interval was selected to capture reasonable resolution for infection stage while also providing sufficient measurements to observe potential differences. Significantly higher morning viral loads were not observed prior to symptom onset in either specimen type in the limited number of specimens collected during this period. However, significantly higher viral loads (*P* < 0.05, Wilcoxon matched-pair signed-rank test) were observed in saliva specimens collected in the morning for the first 16 days of symptomatic infection (Fig. 2C). Differences in *C_T_* values were also significantly lower (*P* < 0.05, Wilcoxon matched-pair signed-rank test) in morning nasal swab specimens from day 4 to day 16 of symptomatic infection (Fig. 2D). Of note, nasal swab viral load appears to increase more quickly to peak than does salivary viral load (Fig. 1A and B), and nasal swabs also achieve higher peak viral loads (Fig. S4C) than does saliva (Fig. S4A); the high rate of increase in viral load in nasal swabs likely obscures subtle daily fluctuations that are more apparent in saliva, where viral load rises more gradually (19). Nasal swabs appear to also be subject to more sampling variability (Fig. S3 and S4D) than saliva (Fig. S2 and S4B), evidenced by *RNase P* control marker *C_T_* values.

### Saliva and nasal swab viral loads in the range of moderate- and low-sensitivity tests underscore utility of morning sampling.

**FIG 3:**
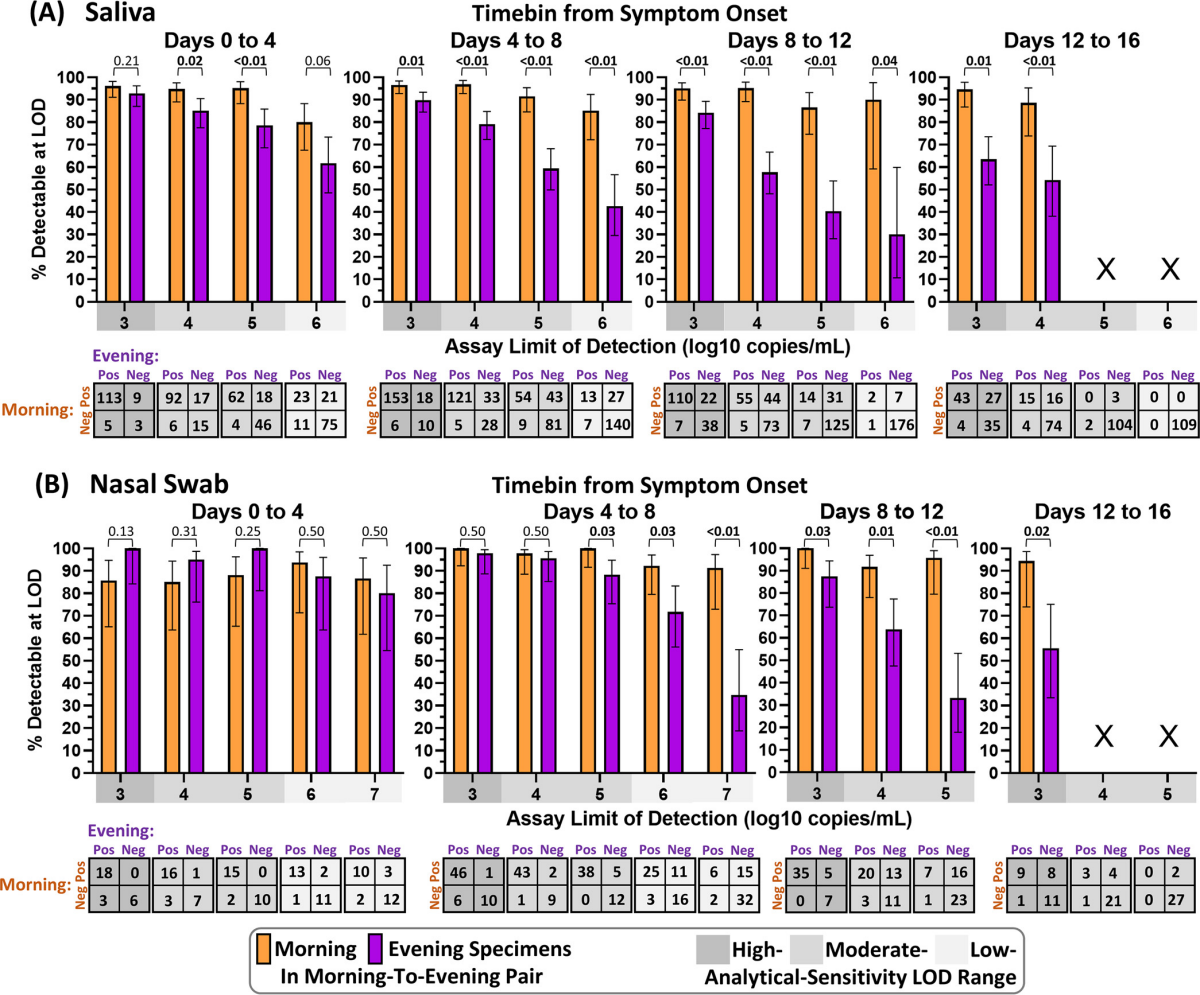
Morning saliva or nasal swab specimen collection yields improved detection across infection stages and assay analytical sensitivities. For each 4-day time bin relative to symptom onset, pairs of sequentially collected morning-to-evening specimens were assessed. In each pair, the viral load in each specimen was used to predict a positive or negative result if tested by an assay with a given limit of detection (LOD) below or above the viral load, respectively. Bar plots show the fraction of pairs with a positive result in either the morning or evening specimen that would be detectable if the morning specimen (orange) or evening specimen (purple) were tested at a given LOD. Error bars indicate the 95% confidence interval. Bars are not shown (X) when fewer than 10 pairs had positive results at the given LOD during the infection time bin. Among LODs and infection time bins with more than 10 positive pairs, the percents detectable for morning versus evening specimens were compared by an upper-tailed McNemar exact test, applied to the 2 × 2 table shown below each comparison. Resulting *P* values are shown above each comparison. Boldfaced values indicate significantly higher detection with morning sampling than with evening sampling. Analysis was performed on saliva specimens (A) and nasal swab specimens (B). Equivalent analysis for evening-to-morning pairs is shown in Fig. S5 in the supplemental material. Pos, positive; Neg, negative.

The observed higher viral loads in specimens collected in the morning upon waking than in those collected later in the day led us to hypothesize that sampling in the morning could detect significantly more infected individuals than sampling in the evening. Because viral loads rise and decline throughout the course of the infection (Fig. 1), we assessed this hypothesis during discrete 4-day time bins following symptom onset. The presymptomatic period was not assessed, as few specimens from this period were available for analysis. Additionally, because COVID-19 diagnostics have analytical sensitivities that span several orders of magnitude, we tested this hypothesis for assays with limits of detection (LODs) of 10^3^, 10^4^, 10^5^, 10^6^, 10^7^ copies/mL; quantitative viral loads measured in each specimen were used to predict whether each specimen would reliably yield a positive result when tested by an assay of each LOD. For each time bin and each LOD, we generated two-by-two matrices to assess the detectability of morning or evening sampling within pairs of sequentially collected morning-to-evening (Fig. 3) specimens. Each time bin and LOD that did not contain at least 10 positive samples from saliva or nasal swab were excluded from this analysis.

For saliva specimens, the advantage of morning sampling was statistically significant in all but two comparisons (Fig. 3A); the two comparisons for which a nonsignificant advantage was observed occurred in the first 4 days of infection, at the LODs of the lowest- and highest-analytical-sensitivity assays (LODs of 10^6^ and 10^3^ copies/mL, respectively). As LOD increases, fewer pairs are predicted to have detectable virus in either the morning or evening specimen; for this reason, confidence intervals widen as the LOD increases, which results in decreased power to detect significant differences in detection by assays with higher LODs. Additionally, assays with lower LODs are able to reliably detect lower viral concentrations, decreasing the impact of fluctuations in viral load from morning to evening sampling on detection.

Morning sampling with nasal swab specimens also exhibited an advantage over evening sampling after 4 days from symptom onset, for all LODs (Fig. 3B). In the first 4 days of infection, a nonsignificant advantage of evening over morning sampling was observed; in this phase of the infection, viral loads in nasal swab specimens typically rise rapidly from undetectable to high (Fig. 1). Therefore, during this rapid rise, the specimen collected later within a pair of successively collected specimens would improve detection; indeed, when morning-to-evening pairs were assessed (Fig. 3), the later (evening) time point had improved detection but when evening-to-morning pairs were assessed (Fig. S5), the later (morning) time point resulted in improved detection.

Similarly, when viral loads are declining, one may expect the earlier time point within a pair of successively collected specimens to exhibit improved detection. We assessed whether this effect was responsible for the improved performance of morning sampling over evening sampling when pairs of successively collected morning-to-evening specimens were compared by performing an equivalent analysis of pairs of successively collected evening-to-morning specimens (Fig. S5). Even with evening-to-morning pairing, morning sampling exhibited an advantage over evening sampling for all comparisons with saliva and nearly all comparisons with nasal swabs. In the three of 12 comparisons where morning sampling with nasal swabs did not exhibit an advantage, two comparisons had equivalent detection by morning or evening sampling, and in the third comparison evening sampling exhibited only a nonsignificant advantage of less than 2% over morning sampling.

This supports that the advantage of morning sampling over evening sampling for both saliva and nasal swabs was robust to whether the morning specimen is collected prior to or following the evening specimen. These results suggest that collecting saliva or nasal swab specimens for SARS-CoV-2 testing in the morning, immediately after waking, can significantly improve detection of symptomatic, infected individuals.

## DISCUSSION

In this study, we quantitatively measured SARS-CoV-2 viral load with high frequency (twice per day) longitudinally through the course of mild COVID-19 infection in saliva for 70 individuals and in nasal swabs for 29 individuals. From these measurements, we identified a pattern of higher viral loads in saliva and nasal swab specimens collected in the morning after waking than in those collected in the evening. Although similar observations have been reported for nasopharyngeal swabs (20, 21), early-morning versus spot oropharyngeal specimens (22), and early-morning saliva versus nasopharyngeal swabs (23) and in wastewater surveillance (24), our study is unique and clinically relevant for three reasons: (i) we measured viral load in specimen types relevant to at-home testing using a high-analytical-sensitivity RT-qPCR assay, which enabled us to infer the performance of diagnostic tests of different analytical sensitivities at each stage of infection; (ii) we collected specimens at high temporal resolution (morning and evening) longitudinally for 2 weeks, starting from early in the course of the infection via prospective sampling of high-risk populations; and (iii) our study provides the largest data set to date that investigates daily patterns in SARS-CoV-2 viral loads, with 1,194 saliva and 661 nasal swab specimens collected longitudinally. From these data, we find compelling evidence that collecting samples for COVID-19 testing in the morning upon waking can significantly improve detection of infected individuals.

The biological and physiological reasons for higher SARS-CoV-2 viral loads in the morning remain unknown but may be due to accumulation of viral material overnight or related to viral replication and immune function. Similar to the improved performance of at-home pregnancy tests with morning urine due to accumulation of human chorionic gonadotropin (25), improved detection of SARS-CoV-2 may be the result of physical accumulation of material (e.g., cells, virions, and nucleic acids) in the upper respiratory tract due to supine positioning (aiding mucociliary clearance) and/or the decreased rate of swallowing at night (26). Higher morning viral loads being due to physical accumulation of nucleic acids is supported by an increased abundance of the constitutive human *RNase P* target in saliva and nasal swab specimens collected in the morning (see Fig. S2A and S3A in the supplemental material). Human salivary production decreases overnight (27), suggesting that higher morning viral loads could be due to a concentration of virus when saliva volume is lower. Given that some individuals exhibit this phenomenon independently of human *RNase P* target abundance (Fig. S2B and S3B), a circadian rhythm in viral replication may also contribute. Regulation and responsiveness of the immune system have been linked to circadian rhythms (28, 29), shown to affect SARS-CoV-2 infection of monocytes in cell culture (30) and proposed as a modulating factor for COVID-19 severity and management (31). Others have proposed cellular interactions between viral proteins and circadian rhythm-dependent host signals (32) and demonstrated circadian rhythm-dependent entry and proliferation of SARS-CoV-2 in lung epithelial cell types in culture (33). Regardless of mechanism, because higher viral loads are associated with replication-competent culturable virus (34, 35), these findings may also suggest a higher risk of transmission in the morning.

As many individuals remain unvaccinated and new variants emerge, it remains critical to identify infections, promptly isolate infected persons, trace and quarantine contacts, and initiate early treatment to improve efficacy. Much of the world lacks access to tests with high analytical sensitivity (36–38). Our findings suggest that strategically collecting specimens in the morning immediately after waking up may improve the performance of available low- to moderate-analytical-sensitivity tests. Morning sampling will not raise the performance of tests with low analytical sensitivity to the levels of those with higher analytical sensitivity; however, even marginal improvements in detection have been shown to reduce deaths from COVID-19 (39).

This study is subject to five main limitations. First, we had a limited number of specimens collected prior to the onset of symptoms, limiting our ability to discern a difference in detectability with morning or evening specimens during the presymptomatic phase of infection. Second, this study was performed prior to the dominance of the Delta and Omicron variants of SARS-CoV-2, which may exhibit different viral load kinetics. Host factors, including vaccination status, may also influence viral load kinetics; nearly all individuals in this cohort were unvaccinated. Third, specimens were self-collected without supervision and thus may have had a different quality from those collected by a health care professional. However, many COVID-19 diagnostics in use utilize self-collected specimens, and measurements of the human *RNase P* gene suggest consistent sampling without failure to collect sufficient material. Fourth, we quantified viral load using RT-qPCR with SARS-CoV-2 *N* gene target. Many COVID-19 diagnostics utilize *N* gene targets, and *N* gene viral loads have been shown to track with other gene targets, suggesting that *N* gene quantification to viral load conversion would be representative to demonstrate a general phenomenon relevant for diagnostics detecting other viral targets. Fifth, this analysis involves inferring positivity by assays with various analytical sensitivities (LODs), based on the quantitatively measured viral loads. A direct comparison with a specific test is needed to test real-world efficacy.

## MATERIALS AND METHODS

### Study design.

Participants were recruited for participation in a COVID-19 household transmission study as previously described (9, 19). Briefly, if at least one member of a household with 2 or more persons had a positive COVID-19 test result within 7 days or was suspected to be positive, all household members aged 6 years and older were eligible to participate. Participants began collecting saliva or saliva and nasal swab specimens on the evening of enrollment and each subsequent morning and evening (as described below). COVID-19-like symptoms were reported via questionnaire with each specimen collection time point.

For participants who were SARS-CoV-2 positive when initially enrolled in the study, symptom onset was defined as the date of first symptoms reported in an enrollment questionnaire. For participants who entered the study SARS-CoV-2 negative but had unrelated symptoms, symptom onset was the first instance of a new COVID-19-like symptom or an increase in symptom severity following their first SARS-CoV-2-positive specimen.

### Specimen collection.

Participants self-collected anterior nares nasal swab and saliva specimens in the Spectrum SDNA-1000 saliva collection kit (Spectrum Solutions LLC, Draper, UT), at home twice per day (after waking up and before going to bed), per manufacturer’s guidelines (although Spectrum devices are not currently authorized for the collection of nasal swab specimens). One participant self-collected both anterior nares nasal swab and saliva specimen in Nest viral transport medium (VTM) (catalog no. NST-NST-202117; Stellar Scientific, Baltimore, MD), and three individuals collected their nasal swab specimens in VTM and their saliva specimens in the Spectrum SDNA-1000 saliva collection kit. Participants were instructed not to ingest anything, smoke, or brush their teeth for at least 30 min prior to collection. For nasal swab collection, participants were asked to gently blow their noses before swabbing (four complete rotations with gentle pressure in each nostril) with sterile flocked swabs. A parent/guardian assisted minors with collection. At collection, participants recorded the date and time and any symptoms experienced in the previous 12 h. Specimens collected between 4 a.m. and 12 p.m. were defined as morning; specimens collected between 3 p.m. and 3 a.m. were defined as evening (see Fig. S1 in the supplemental material).

### Cohort of individuals with SARS-CoV-2 infection.

Between September 2020 and April 2021, 72 participants from 39 households in southern California had acute SARS-CoV-2 infection. Of these, 2 never reported experiencing symptoms and were not included in subsequent analyses where viral loads are aligned with date of symptom onset. Of the 70 symptomatic individuals from 37 households included in the analyses (Table 1), all 70 collected saliva specimens while a subset of 29 individuals collected both saliva and nasal swab specimens every morning and every evening while enrolled, from which we quantified viral loads.

**TABLE 1:** Demographic and medical information on participants^*a*^

Demographic or medical characteristic	Participants contributing specimen type(s):			
	Saliva (*n* = 70)		Saliva and nasal swabs (*n* = 29)	
	No.	%	No.	%
Sex^*b*^				
Male	25	35.7	9	31.0
Female	45	64.3	20	69.0
Age, yr				
6–11	6	8.6	1	3.4
12–17	9	12.9	4	13.8
18–24	9	12.9	3	10.3
25–35	17	24.3	10	34.5
36–45	12	17.1	3	10.3
46–55	11	15.7	6	20.7
56–65	5	7.1	2	6.9
65+	1	1.4	0	0.0
Race				
Asian/Pacific Islander	6	8.6	2	6.9
Black/African American	2	2.9	2	6.9
Native American	0	0.0	0	0.0
White	33	47.1	15	51.7
Multiple races	4	5.7	3	10.3
Other/unknown^*c*^	25	35.7	7	24.1
Ethnicity				
Hispanic	52	74.3	21	72.4
Non-Hispanic	17	24.3	8	27.6
Unknown	1	1.4	0	0.0
Tobacco smoker or vape user history				
Current	5	7.1	3	10.3
Former	15	21.4	9	31.0
Never	43	61.4	16	55.2
Unknown	7	10.0	1	3.4
Active medications and supplements				
Vitamins/supplements	47	67.1	21	72.4
Acetaminophen/NSAIDs^*d*^	33	47.1	13	44.8
Allergy medications/antihistamines	11	15.7	3	10.3
Antibiotics/antivirals	3	4.3	0	0.0
Steroid drug	3	4.3	1	3.4
Medical comorbidities				
Asthma	6	8.6	1	3.4
Anxiety or depression	4	5.7	2	6.9
Diabetes	4	5.7	3	10.3
Obesity	4	5.7	2	6.9
Hypertension	3	4.3	1	3.4
Immunocompromise	0	0.0	0	0.0
SARS-CoV-2 vaccination status^*e*^				
Partially vaccinated	1	1.4	1	3.4
Completed vaccination	0	0.0	0	0.0
No vaccines reported	69	98.6	28	96.6

a Demographic and medical information on participants was collected via online questionnaire upon study enrollment. All participants (*n* = 70) collected saliva; of these 70 individuals, 29 additionally collected nasal swabs.

b Participants were asked to report both sex assigned at birth and current gender identity; all 70 participants in this cohort reported cisgender identities.

c A total of 10 individuals contributing saliva and 2 individuals contributing both saliva and nasal swabs indicated “Other” in the race field and wrote in “Hispanic” or “Latinx/Latina/Latino.”

d NSAIDs, nonsteroidal anti-inflammatory drugs. This number includes 2 individuals (each contributing both saliva and nasal swabs) who indicated they were taking a pain-relieving medication but did not specify the type.

e Complete vaccination was considered a two-dose series of Pfizer-BioNTech (COMIRNATY) or Moderna (Spikevax) COVID-19 vaccines or one dose of Johnson & Johnson’s Janssen COVID-19 vaccine.

Individuals were enrolled at various stages of infection. Of the 70 infected, symptomatic individuals, 58 were positive for SARS-CoV-2 in the first saliva or saliva and nasal swab specimen collected upon enrollment while 12 were initially negative but became positive while enrolled in the study; of these 12 individuals, 7 were collecting both saliva and nasal swabs, and the viral loads and symptoms of these individuals have been previously reported (6). Of the 58 cases positive on enrollment, 50 (86.2%) were already experiencing mild COVID-19-like symptoms and 8 (13.8%) were presymptomatic. Of the 20 individuals who were either presymptomatic (8) or negative for SARS-CoV-2 (12) on enrollment, COVID-19 symptom onset occurred an average of 1.2 days after the first SARS-CoV-2-positive saliva specimen.

The mean age of the saliva cohort was 32.8 years (standard deviation [SD], ±16.0 years), and the mean age was 33.9 years (SD, ±15.2 years) among those collecting both saliva and nasal swabs. Health conditions and medications that may have impacted viral load kinetics are provided for individual participants in the supplemental material. No participants required hospitalization. At the time of these participants’ enrollment in the study (September 2020 to April 2021), vaccines were either unavailable or limited to priority groups. Only one individual (Fig. S2H and S3H) reported receiving a COVID-19 vaccine (first dose of Pfizer-BioNTech COVID-19, ~3 weeks before enrollment).

### Extraction and quantification of viral load by RT-qPCR.

Specimen processing was performed as previously described (9). Briefly, 400 or 200 μL of fluid from each saliva or nasal swab specimen, respectively, was extracted using the MagMAX Viral/Pathogen nucleic acid isolation kit (ThermoFisher Scientific; catalog no. A42352), followed by the CDC 2019-novel coronavirus (2019-nCoV) real-time RT-PCR diagnostic panel, which targets the SARS-CoV-2 *N1* and *N2* genes, as well as a human *RNase P* control. *N1* gene *C_T_* values were converted to viral load using an equation derived from a standard curve of heat-inactivated SARS-CoV-2 particles spiked into human specimen matrix validated previously by independent RT-double differential PCR (ddPCR) measurement (6).

### Statistical analyses.

Initial processing was performed in Python v3.8.2, with calculation of log-transformed averages (Fig. 1). Data were exported, and differences in *C_T_* from sequential specimens were calculated in Microsoft Excel (Fig. 2A to D). Plots were prepared in GraphPad Prism 9.2.0, including calculation of medians (Fig. 2). For comparison of the differences between morning and evening viral loads and differences in *C_T_* values, the Wilcoxon matched-pair signed-rank test was performed using GraphPad (Fig. 2). An upper-tailed McNemar test to compare inferred percentages of infections detectable by assays with various LODs for specimens collected in the morning or evening (Fig. 3 and Fig. S5) was performed in Python v3.8.2 using the scipy.stats package (40).

### Data availability.

The data underlying the results presented in the study are available at CaltechDATA at https://data.caltech.edu/records/20049.

## References

[1] Wadhwa A, Fisher KA, Silver R, Koh M, Arons MM, Miller DA, McIntyre AF, Vuong JT, Kim K, Takamiya M, Binder AM, Tate JE, Armstrong PA, Black SR, Mennella CC, Levin R, Gubser J, Jones B, Welbel SF, Moonan PK, Curran K, Ghinai I, Doshi R, Zawitz CJ. 2021. Identification of presymptomatic and asymptomatic cases using cohort-based testing approaches at a large correctional facility—Chicago, Illinois, USA, May 2020. Clin Infect Dis 72:e128–e135. doi:10.1093/cid/ciaa1802. 33270101 PMC7799274

[2] Dora AV, Winnett A, Jatt LP, Davar K, Watanabe M, Sohn L, Kern HS, Graber CJ, Goetz MB. 2020. Universal and serial laboratory testing for SARS-CoV-2 at a long-term care skilled nursing facility for veterans—Los Angeles, California, 2020. MMWR Morb Mortal Wkly Rep 69:651–655. doi:10.15585/mmwr.mm6921e1. 32463809 PMC7269604

[3] Lai J, German J, Hong F, Sheldon Tai S-H, McPhaul KM, Milton DK, for the University of Maryland StopCOVID Research Group. 2021. Comparison of saliva and mid-turbinate swabs for detection of COVID-19. medRxiv 2021.2012.2001.21267147. doi:10.1101/2021.12.01.21267147.

[4] Wyllie AL, Fournier J, Casanovas-Massana A, Campbell M, Tokuyama M, Vijayakumar P, Warren JL, Geng B, Muenker MC, Moore AJ, Vogels CBF, Petrone ME, Ott IM, Lu P, Venkataraman A, Lu-Culligan A, Klein J, Earnest R, Simonov M, Datta R, Handoko R, Naushad N, Sewanan LR, Valdez J, White EB, Lapidus S, Kalinich CC, Jiang X, Kim DJ, Kudo E, Linehan M, Mao T, Moriyama M, Oh JE, Park A, Silva J, Song E, Takahashi T, Taura M, Weizman O-E, Wong P, Yang Y, Bermejo S, Odio CD, Omer SB, Dela Cruz CS, Farhadian S, Martinello RA, Iwasaki A, Grubaugh ND, Ko AI. 2020. Saliva or nasopharyngeal swab specimens for detection of SARS-CoV-2. N Engl J Med 383:1283–1286. doi:10.1056/NEJMc2016359. 32857487 PMC7484747

[5] Ke R, Martinez PP, Smith RL, Gibson LL, Mirza A, Conte M, Gallagher N, Luo CH, Jarrett J, Conte A, Liu T, Farjo M, Walden KKO, Rendon G, Fields CJ, Wang L, Fredrickson R, Edmonson DC, Baughman ME, Chiu KK, Choi H, Scardina KR, Bradley S, Gloss SL, Reinhart C, Yedetore J, Quicksall J, Owens AN, Broach J, Barton B, Lazar P, Heetderks WJ, Robinson ML, Mostafa HH, Manabe YC, Pekosz A, McManus DD, Brooke CB. 2021. Daily sampling of early SARS-CoV-2 infection reveals substantial heterogeneity in infectiousness. medRxiv. doi:10.1101/2021.07.12.21260208. PMC9084242 35484231

[6] Wölfel R, Corman VM, Guggemos W, Seilmaier M, Zange S, Müller MA, Niemeyer D, Jones TC, Vollmar P, Rothe C, Hoelscher M, Bleicker T, Brünink S, Schneider J, Ehmann R, Zwirglmaier K, Drosten C, Wendtner C. 2020. Virological assessment of hospitalized patients with COVID-2019. Nature 581:465–469. doi:10.1038/s41586-020-2196-x. 32235945

[7] Smith RL, Gibson LL, Martinez PP, Ke R, Mirza A, Conte M, Gallagher N, Conte A, Wang L, Fredrickson R, Edmonson DC, Baughman ME, Chiu KK, Choi H, Jensen TW, Scardina KR, Bradley S, Gloss SL, Reinhart C, Yedetore J, Owens AN, Broach J, Barton B, Lazar P, Henness D, Young T, Dunnett A, Robinson ML, Mostafa HH, Pekosz A, Manabe YC, Heetderks WJ, McManus DD, Brooke CB. 2021. Longitudinal assessment of diagnostic test performance over the course of acute SARS-CoV-2 infection. medRxiv. https://www.medrxiv.org/content/10.1101/2021.03.19.21253964v2. 10.1093/infdis/jiab337 PMC8448437 34191025

[8] Singanayagam A, Hakki S, Dunning J, Madon KJ, Crone MA, Koycheva A, Derqui-Fernandez N, Barnett JL, Whitfield MG, Varro R, Charlett A, Kundu R, Fenn J, Cutajar J, Quinn V, Conibear E, Barclay W, Freemont PS, Taylor GP, Ahmad S, Zambon M, Ferguson NM, Lalvani A, Badhan A, Dustan S, Tejpal C, Ketkar AV, Narean JS, Hammett S, McDermott E, Pillay T, Houston H, Luca C, Samuel J, Bremang S, Evetts S, Poh J, Anderson C, Jackson D, Miah S, Ellis J, Lackenby A. 2022. Community transmission and viral load kinetics of the SARS-CoV-2 delta (B.1.617.2) variant in vaccinated and unvaccinated individuals in the UK: a prospective, longitudinal, cohort study. Lancet Infect Dis 22:183–195. doi:10.1016/S1473-3099(21)00648-4. 34756186 PMC8554486

[9] Savela ES, Viloria Winnett A, Romano AE, Porter MK, Shelby N, Akana R, Ji J, Cooper MM, Schlenker NW, Reyes JA, Carter AM, Barlow JT, Tognazzini C, Feaster M, Goh Y-Y, Ismagilov RF. 2022. Quantitative SARS-CoV-2 viral-load curves in paired saliva and nasal swabs inform appropriate respiratory sampling site and analytical test sensitivity required for earliest viral detection. J Clin Microbiol 60:e01785-21. doi:10.1128/jcm.01785-21. 34911366 PMC8849374

[10] Kissler SM, Fauver JR, Mack C, Tai CG, Breban MI, Watkins AE, Samant RM, Anderson DJ, Metti J, Khullar G, Baits R, MacKay M, Salgado D, Baker T, Dudley JT, Mason CE, Ho DD, Grubaugh ND, Grad YH. 2021. Viral dynamics of SARS-CoV-2 variants in vaccinated and unvaccinated persons. N Engl J Med 385:2489–2491. doi:10.1056/NEJMc2102507. 34941024 PMC8693673

[11] Kissler SM, Fauver JR, Mack C, Olesen SW, Tai C, Shiue KY, Kalinich CC, Jednak S, Ott IM, Vogels CBF, Wohlgemuth J, Weisberger J, DiFiori J, Anderson DJ, Mancell J, Ho DD, Grubaugh ND, Grad YH. 2021. Viral dynamics of acute SARS-CoV-2 infection and applications to diagnostic and public health strategies. PLoS Biol 19:e3001333. doi:10.1371/journal.pbio.3001333. 34252080 PMC8297933

[12] FDA. 2020. SARS-CoV-2 reference panel comparative data. FDA, Silver Spring, MD. https://www.fda.gov/medical-devices/coronavirus-covid-19-and-medical-devices/sars-cov-2-reference-panel-comparative-data.

[13] Brümmer LE, Katzenschlager S, Gaeddert M, Erdmann C, Schmitz S, Bota M, Grilli M, Larmann J, Weigand MA, Pollock NR, Macé A, Carmona S, Ongarello S, Sacks JA, Denkinger CM. 2021. Accuracy of novel antigen rapid diagnostics for SARS-CoV-2: a living systematic review and meta-analysis. PLoS Med 18:e1003735. doi:10.1371/journal.pmed.1003735. 34383750 PMC8389849

[14] WHO. 2020. Global partnership to make available 120 million affordable, quality COVID-19 rapid tests for low- and middle-income countries. WHO, Geneva, Switzerland. https://www.who.int/news/item/28-09-2020-global-partnership-to-make-available-120-million-affordable-quality-covid-19-rapid-tests-for-low--and-middle-income-countries.

[15] Connor A, Hariharan N, Carson S, Sanders KC, Vosburg KB, Sabot O. 27 October 2021. Access to COVID-19 testing in low- and middle-income countries is still critical to achieving health equity. Health Affairs Blog. https://www.healthaffairs.org/do/10.1377/forefront.20211026.483412#:~:text=Health%20Affairs%20Forefront-,Access%20To%20COVID%2D19%20Testing%20In%20Low%2D%20And%20Middle%2D,Critical%20To%20Achieving%20Health%20Equity&text=The%20world%20has%20witnessed%20the,%2C%20economies%2C%20and%20health%20systems.

[16] PATH. 2022. Global availability of COVID-19 diagnostic tests. PATH, Seattle, WA. https://www.path.org/programs/diagnostics/covid-dashboard-global-availability-covid-19-diagnostic-tests/.

[17] Borrmann H, McKeating JA, Zhuang X. 2021. The circadian clock and viral infections. J Biol Rhythms 36:9–22. doi:10.1177/0748730420967768. 33161818 PMC7924106

[18] Diallo AB, Coiffard B, Leone M, Mezouar S, Mege J-L. 2020. For whom the clock ticks: clinical chronobiology for infectious diseases. Front Immunol 11:1457. doi:10.3389/fimmu.2020.01457. 32733482 PMC7363845

[19] Winnett A, Cooper MM, Shelby N, Romano AE, Reyes JA, Ji J, Porter MK, Savela ES, Barlow JT, Akana R, Tognazzini C, Feaster M, Goh Y-Y, Ismagilov RF. 2020. SARS-CoV-2 viral load in saliva rises gradually and to moderate levels in some humans. medRxiv 2020.2012.2009.20239467. doi:10.1101/2020.12.09.20239467.

[20] McNaughton CD, Adams NM, Hirschie Johnson C, Ward MJ, Schmitz JE, Lasko TA. 2021. Diurnal variation in SARS-CoV-2 PCR test results: test accuracy may vary by time of day. J Biol Rhythms 36:595–601. doi:10.1177/07487304211051841. 34696614 PMC8599649

[21] Liu M, Li Q, Zhou J, Ai W, Zheng X, Zeng J, Liu Y, Xiang X, Guo R, Li X, Wu X, Xu H, Jiang L, Zhang H, Chen J, Tian L, Luo J, Luo C. 2020. Value of swab types and collection time on SARS-COV-2 detection using RT-PCR assay. J Virol Methods 286:113974. doi:10.1016/j.jviromet.2020.113974. 32949663 PMC7493793

[22] Hung DL-L, Li X, Chiu KH-Y, Yip CC-Y, To KK-W, Chan JF-W, Sridhar S, Chung TW-H, Lung K-C, Liu RW-T, Kwan GS-W, Hung IF-N, Cheng VC-C, Yuen K-Y. 2020. Early-morning vs spot posterior oropharyngeal saliva for diagnosis of SARS-CoV-2 infection: implication of timing of specimen collection for community-wide screening. Open Forum Infect Dis 7:ofaa210. doi:10.1093/ofid/ofaa210. 32577428 PMC7299521

[23] Rao M, Rashid FA, Sabri FSAH, Jamil NN, Zain R, Hashim R, Amran F, Kok HT, Samad MAA, Ahmad N. 2021. Comparing nasopharyngeal swab and early morning saliva for the identification of severe acute respiratory syndrome coronavirus 2 (SARS-CoV-2). Clin Infect Dis 72:e352–e356. doi:10.1093/cid/ciaa1156. 32761244 PMC7454325

[24] Bivins A, North D, Wu Z, Shaffer M, Ahmed W, Bibby K. 2021. Within- and between-day variability of SARS-CoV-2 RNA in municipal wastewater during periods of varying COVID-19 prevalence and positivity. ACS ES T Water 1:2097–2108. doi:10.1021/acsestwater.1c00178.

[25] FDA. 2019. Home use tests: pregnancy. FDA, Silver Spring, MD. https://www.fda.gov/medical-devices/home-use-tests/pregnancy.

[26] Uludag IF, Tiftikcioglu BI, Ertekin C. 2016. Spontaneous swallowing during all-night sleep in patients with Parkinson disease in comparison with healthy control subjects. Sleep 39:847–854. doi:10.5665/sleep.5640. 26943467 PMC4791618

[27] Dawes C. 1972. Circadian rhythms in human salivary flow rate and composition. J Physiol 220:529–545. doi:10.1113/jphysiol.1972.sp009721. 5016036 PMC1331668

[28] Mazzoccoli G, Vinciguerra M, Carbone A, Relógio A. 2020. The circadian clock, the immune system, and viral infections: the intricate relationship between biological time and host-virus interaction. Pathogens 9:83. doi:10.3390/pathogens9020083. 32012758 PMC7168639

[29] Niemeyer JE. 2016. Viruses and circadian rhythms. Lab Anim (NY) 46:7. doi:10.1038/laban.1176. 27991877

[30] Diallo AB, Gay L, Coiffard B, Leone M, Mezouar S, Mege J-L. 2021. Daytime variation in SARS-CoV-2 infection and cytokine production. Microb Pathog 158:105067. doi:10.1016/j.micpath.2021.105067. 34175433 PMC8225298

[31] Ray S, Reddy AB. 2020. COVID-19 management in light of the circadian clock. Nat Rev Mol Cell Biol 21:494–495. doi:10.1038/s41580-020-0275-3. 32699357 PMC7374068

[32] Meira e Cruz M, Miyazawa M, Gozal D. 2020. Putative contributions of circadian clock and sleep in the context of SARS-CoV-2 infection. Eur Respir J 55:2001023. doi:10.1183/13993003.01023-2020. 32350105 PMC7191115

[33] Zhuang X, Tsukuda S, Wrensch F, Wing PAC, Schilling M, Harris JM, Borrmann H, Morgan SB, Cane JL, Mailly L, Thakur N, Conceicao C, Sanghani H, Heydmann L, Bach C, Ashton A, Walsh S, Tan TK, Schimanski L, Huang K-YA, Schuster C, Watashi K, Hinks TSC, Jagannath A, Vausdevan SR, Bailey D, Baumert TF, McKeating JA. 2021. The circadian clock component BMAL1 regulates SARS-CoV-2 entry and replication in lung epithelial cells. iScience 24:103144. doi:10.1016/j.isci.2021.103144. 34545347 PMC8443536

[34] Gallichotte EN, Quicke KM, Sexton NR, Fitzmeyer E, Young MC, Janich AJ, Dobos K, Pabilonia KL, Gahm G, Carlton EJ, Ebel GD, Ehrhart N. 2020. Longitudinal surveillance for SARS-CoV-2 among staff in six Colorado long-term care facilities: epidemiologic, virologic and sequence analysis. medRxiv. doi:10.1101/2020.06.08.20125989. PMC8579921 34756092

[35] Perera RAPM, Tso E, Tsang OTY, Tsang DNC, Fung K, Leung YWY, Chin AWH, Chu DKW, Cheng SMS, Poon LLM, Chuang VWM, Peiris M. 2020. SARS-CoV-2 virus culture and subgenomic RNA for respiratory specimens from patients with mild coronavirus disease. Emerg Infect Dis 26:2701–2704. doi:10.3201/eid2611.203219. 32749957 PMC7588524

[36] Okeke IN, Ihekweazu C. 2021. The importance of molecular diagnostics for infectious diseases in low-resource settings. Nat Rev Microbiol 19:547–548. doi:10.1038/s41579-021-00598-5. 34183821 PMC8237771

[37] Sander A-L, Yadouleton A, Moreira-Soto A, Tchibozo C, Hounkanrin G, Badou Y, Fischer C, Krause N, Akogbeto P, de Oliveira Filho EF, Dossou A, Brünink S, Drosten C, Aïssi MAJ, Harouna Djingarey M, Hounkpatin B, Nagel M, Drexler JF. 2021. An observational laboratory-based assessment of SARS-CoV-2 molecular diagnostics in Benin, Western Africa. mSphere 6:e00979-20. doi:10.1128/mSphere.00979-20. 33441410 PMC7845609

[38] The Lancet Global Health. 2021. Essential diagnostics: mind the gap. Lancet Glob Health 9:e1474. doi:10.1016/S2214-109X(21)00467-8. 34678179 PMC8654090

[39] Terriau A, Albertini J, Montassier E, Poirier A, Le Bastard Q. 2021. Estimating the impact of virus testing strategies on the COVID-19 case fatality rate using fixed-effects models. Sci Rep 11:21650. doi:10.1038/s41598-021-01034-7. 34737362 PMC8569180

[40] Virtanen P, Gommers R, Oliphant TE, Haberland M, Reddy T, Cournapeau D, Burovski E, Peterson P, Weckesser W, Bright J, van der Walt SJ, Brett M, Wilson J, Millman KJ, Mayorov N, Nelson ARJ, Jones E, Kern R, Larson E, Carey CJ, Polat İ, Feng Y, Moore EW, VanderPlas J, Laxalde D, Perktold J, Cimrman R, Henriksen I, Quintero EA, Harris CR, Archibald AM, Ribeiro AH, Pedregosa F, van Mulbregt P, SciPy 1.0 Contributors. 2020. SciPy 1.0: fundamental algorithms for scientific computing in Python. Nat Methods 17:261–272. doi:10.1038/s41592-019-0686-2. 32015543 PMC7056644

