# Supplementary material for: Morning SARS-CoV-2 Testing Yields Better Detection of Infection Due to Higher Viral Loads in Saliva and Nasal Swabs upon Waking
Source: Microbiol Spectr. 2022 Oct 26;10(6):e03873-22. doi: 10.1128/spectrum.03873-22 (PMC9769854; doi:10.1128/spectrum.03873-22)
Supplement: Supplemental file 1 — Supplemental material. Download spectrum.03873-22-s0001.pdf, PDF file, 2.3 MB [file spectrum.03873-22-s0001.pdf]

Supporting Information for:

Morning SARS-CoV-2 testing yields better detection of infection due to higher viral loads in saliva and nasal-swabs upon waking

Alexander Viloria Winnett[1«], Michael K. Porter[1«], Anna E. Romano[1], Emily S. Savela[1], Reid Akana[1], Natasha Shelby[1], Jessica A. Reyes[1], Noah W. Schlenker[1], Matthew M. Cooper[1], Alyssa M. Carter[1], Jenny Ji[1], Jacob T. Barlow[1], Colten Tognazzini[2], Matthew Feaster[2], Ying-Ying Goh[2], Rustem F. Ismagilov[1]

1. California Institute of Technology, 1200 E. California Blvd., Pasadena, CA, USA 91125
2. City of Pasadena Public Health Department, 1845 N. Fair Oaks Ave., Pasadena, CA, USA 91103

« These authors contributed equally to this report.

*Correspondence to: rustem.admin@caltech.edu

Contents:

Additional Specimen Details from Figure 1
Supplementary Figures S1-S4
Author Contribution Statements

ADDITIONAL PARTICIPANT AND SPECIMEN DETAILS FROM FIGURE 1

Viral load was quantified from an average of 32 saliva specimens (SD ±6 specimens) each from the 12 participants in the negative-on-enrollment cohort, while on average 13 saliva specimens (SD ±10 specimens) each were processed from 58 participants positive-on-enrollment (**Figure S2**). For nasal swabs, an average of 35 specimens (SD ±7 specimens) were quantified from 7 participants in the negative-on-enrollment cohort, while viral load was quantified in an average of 17 nasal-swab specimens (SD ±9 specimens) from 22 participants who were positive-on-enrollment (**Figure S3**).

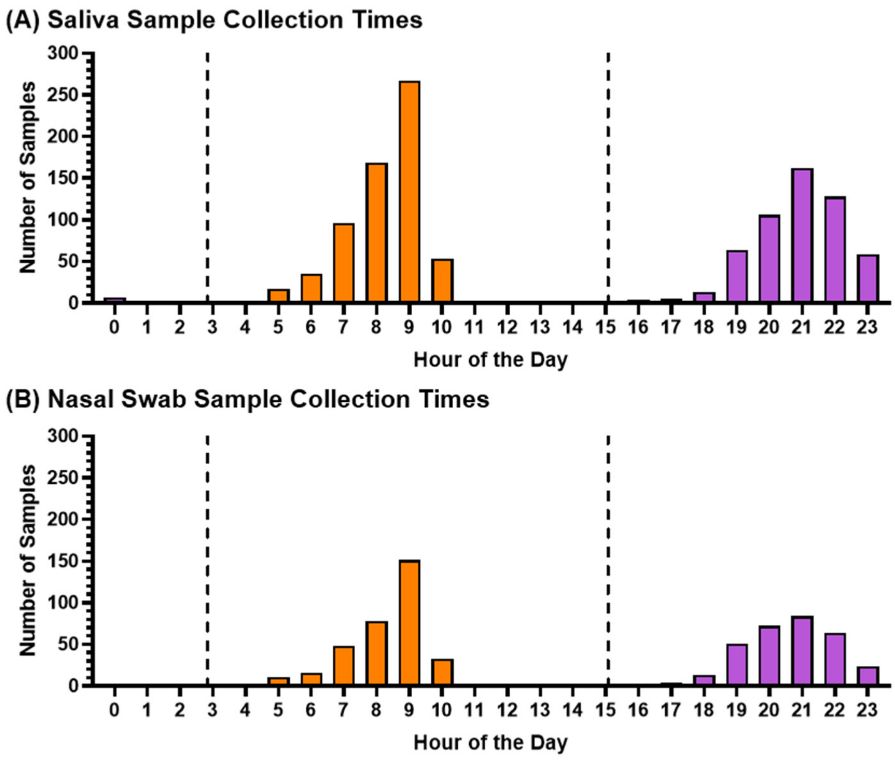

Figure S1. Frequency of Saliva and Nasal-Swab Specimen Collection Times. Study participants either collected saliva only, or saliva then anterior nasal swab at the same time point, and were instructed to collect specimens immediately after waking up and immediately before bed (see Methods for detailed instructions). The frequency of specimens collected by each hour of the day is plotted for 1194 saliva specimens **(A)** and 661 nasal-swab specimens **(B)**. Dashed vertical line indicates cutoff for morning (3 AM to 12 PM) and evening (3 PM to 3 AM) collected specimens used in this study.

[figure on next page] **Figure S2. Individual salivary RT-PCR Ct measurements, for SARS-CoV-2 *N1* gene target (red) and human *RNase P* control gene target (black), relative to symptom onset.** Matching panel labels correspond to the same participant shown in Figure S3. Underlined panel labels indicate that the participant converted from SARS-CoV-2-negative to -positive while enrolled in the study. Grey dashed line indicates Ct threshold for positivity. ND indicates Not Detected.

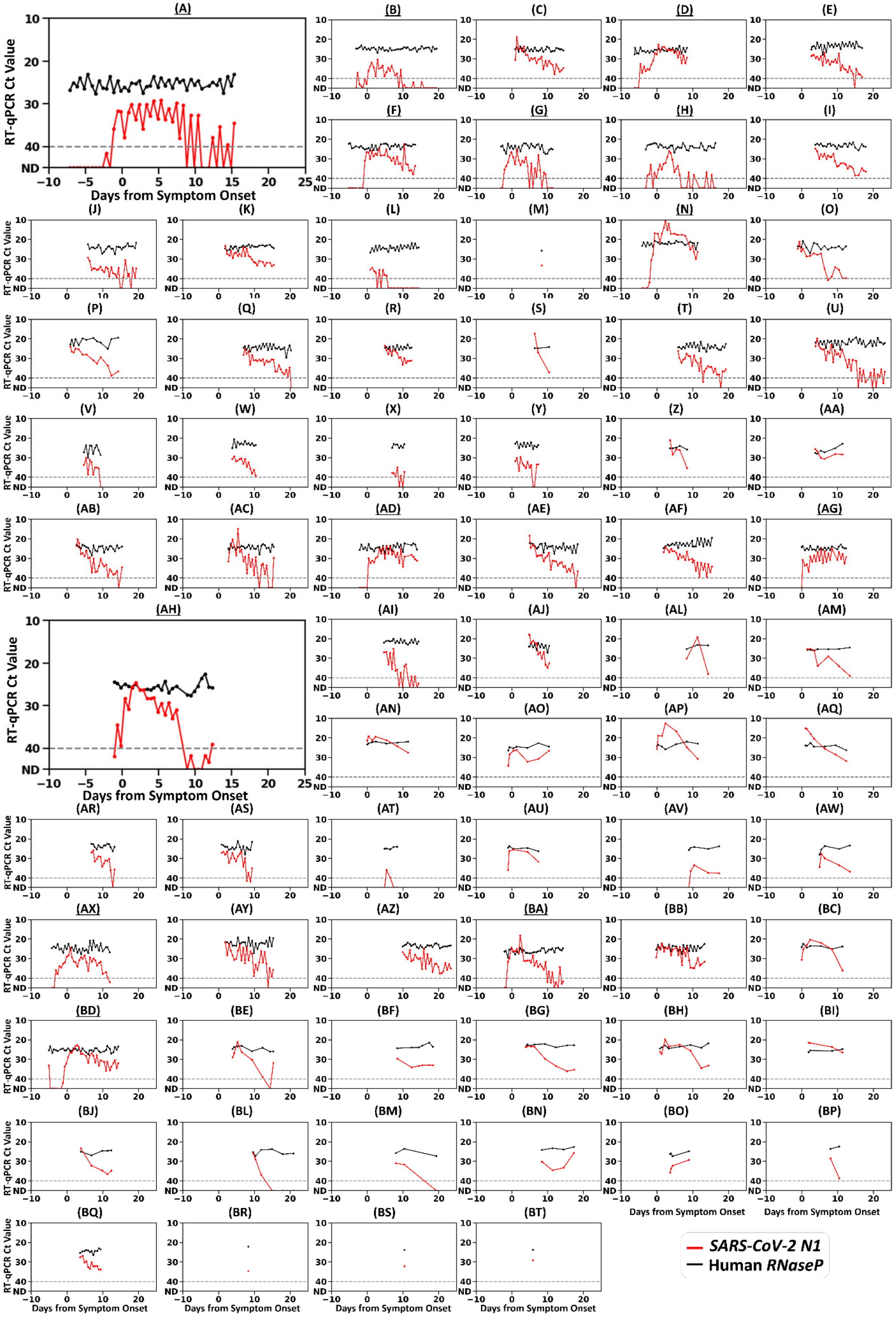
(A)
(B)
(C)
(D)
(E)
(F)
(G)
(H)
(I)
(J)
(K)
(L)
(M)
(N)
(O)
(P)
(Q)
(R)
(S)
(T)
(U)
(V)
(W)
(X)
(Y)
(Z)
(AA)
(AB)
(AC)
(AD)
(AE)
(AF)
(AG)
(AH)
(AI)
(AJ)
(AL)
(AM)
(AN)
(AO)
(AP)
(AQ)
(AR)
(AS)
(AT)
(AU)
(AV)
(AW)
(AX)
(AY)
(AZ)
(BA)
(BB)
(BC)
(BD)
(BE)
(BF)
(BG)
(BH)
(BI)
(BJ)
(BL)
(BM)
(BN)
(BO)
(BP)
(BQ)
(BR)
(BS)
(BT)
RT-qPCR Ct Value
Days from Symptom Onset
10
20
30
40
ND
−10
−5
0
5
10
15
20
25
SARS-CoV-2 N1
Human RNaseP

Of 70 individuals providing saliva specimens, 58 reported good, very good, or excellent health status. While enrolled, a total of 47 participants were taking vitamins and/or supplements, 33 were taking acetaminophen or an NSAID, 11 participants were taking an antihistamine and/or allergy medication, 2 participants were taking a statin (**Fig S2O, Fig S2U**), 2 participants were taking both an antibiotic and anti-seizure medication (**Fig S2AR, Fig S2AN**), and 3 individuals were taking a steroid drug (2 taking the inhaled medication fluticasone [**Fig S2N, Fig S2BP**], and 1 taking prednisone [**Fig S2BS**]). One participant (**Fig S2BT**) with Hepatitis C and HIV infection was taking Biktarvy (bictegravir/emtricitabine/tenofovir alafenamide). Additionally, one participant was pregnant (**Fig S2AB**).

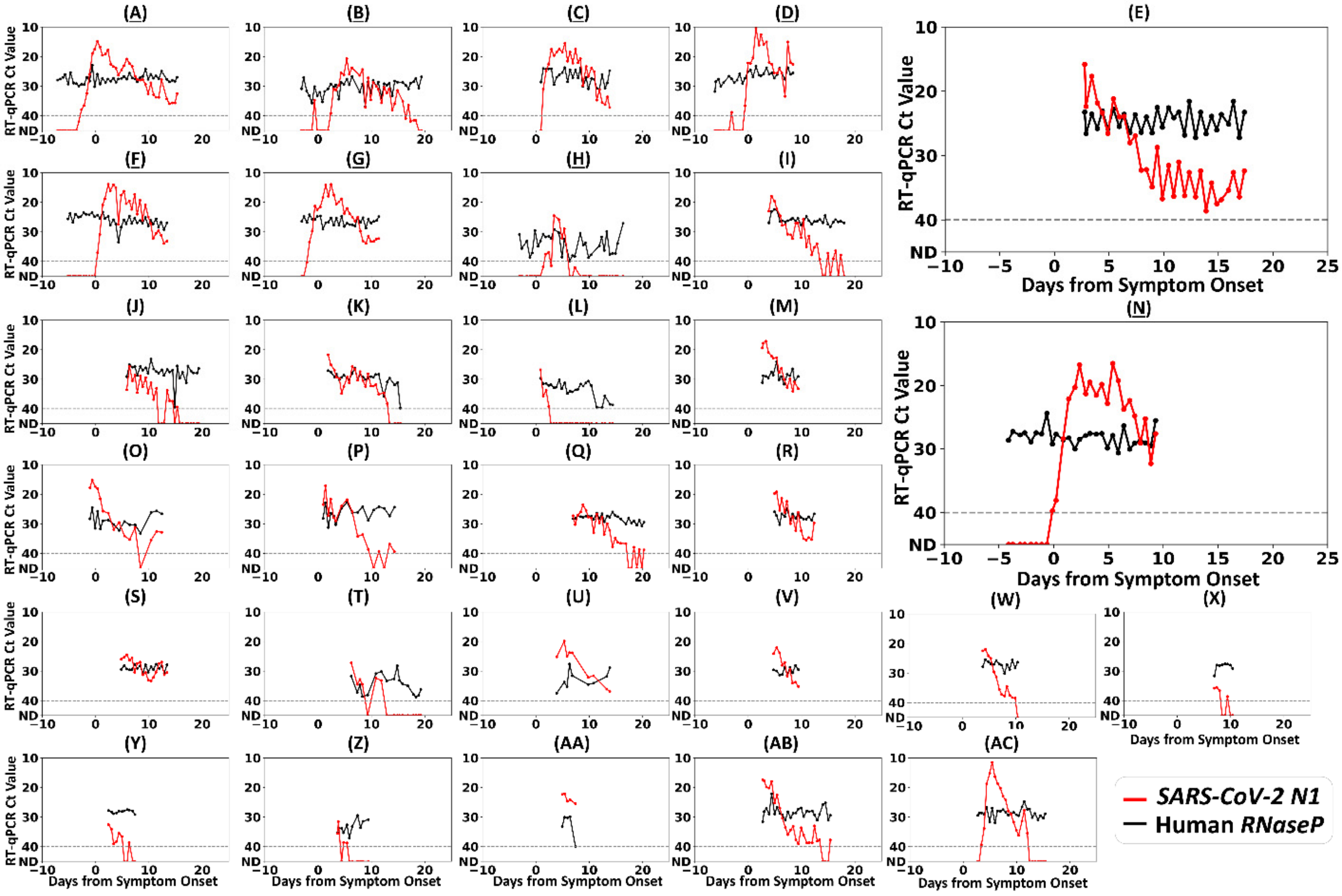

Figure S3. Individual nasal-swab RT-PCR Ct measurements, for SARS-CoV-2 *N1* gene target and human *RNase P* control gene target. Each panel shows the measured *SARS-CoV-2 N1* Ct values (red), and human *RNase P* Ct values (black) for an individual participant, relative to symptom onset. Matching panel labels correspond to the same participant shown in Figure S2. Underlined panel labels indicate that the participant converted from SARS-CoV-2-negative to -positive while enrolled in the study. Grey dashed line indicates Ct threshold for positivity. ND indicates Not Detected.

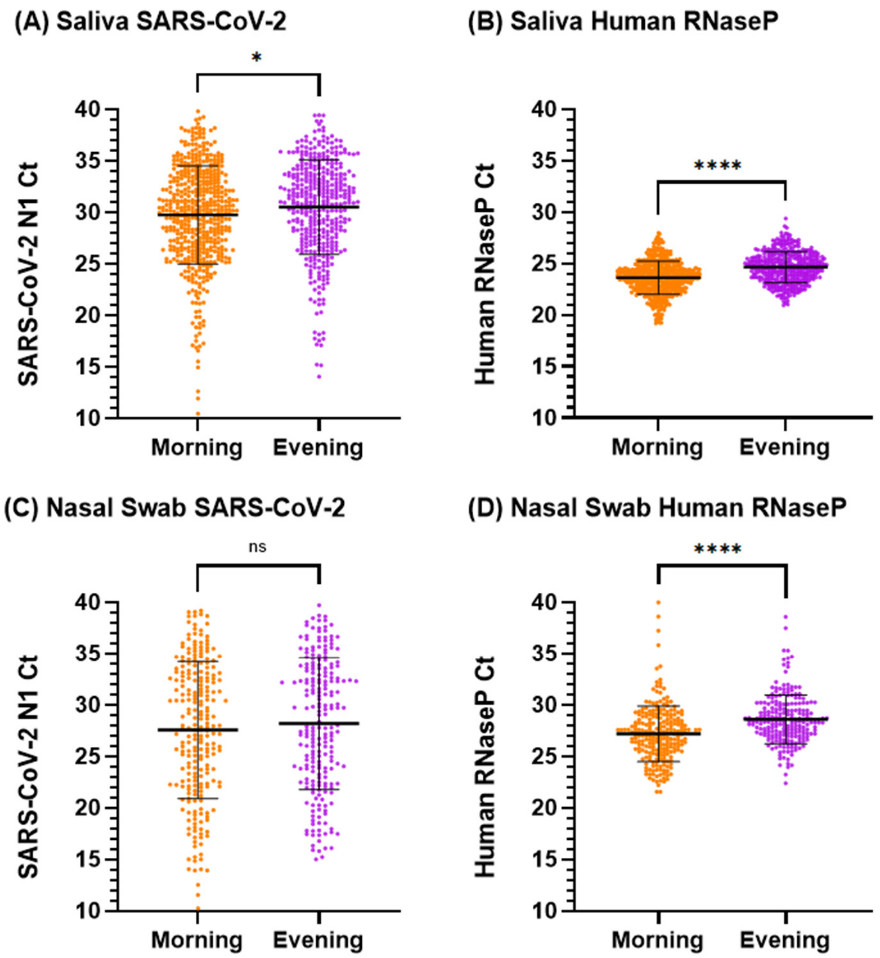

Figure S4. Aggregated SARS-CoV-2 *N1* and human *RNase P* Ct values grouped by specimens collected in the morning and evening. A) Direct comparison of aggregated Ct values for *SARS-CoV-2 N1* gene target, measured from all SARS-CoV-2 positive saliva specimens from all participants, by either morning or evening collection time **B)** Direct comparison of aggregated Ct values for human *RNase P* target from all SARS-CoV-2 positive saliva specimens from all participants, by either morning or evening collection time **C)** Direct comparison of aggregated Ct values for *SARS-CoV-2 N1* gene, measured from all SARS-CoV-2 positive nasal-swab specimens from all participants, by either morning or evening collection time **D)** Direct comparison of aggregated Ct values for human *RNase P* target from all SARS-CoV-2 positive nasal-swab specimens from all participants, by either morning or evening collection time. Specimens with morning collection times are shown as orange points, while evening are shown as purple points. Black lines indicate mean Ct value, with error bars representing standard deviation. Statistical comparison of Ct values for groups performed by unpaired t-test without correction: ns indicates nonsignificant difference, * indicates $P < 0.05$, **** indicates $P < 0.001$.

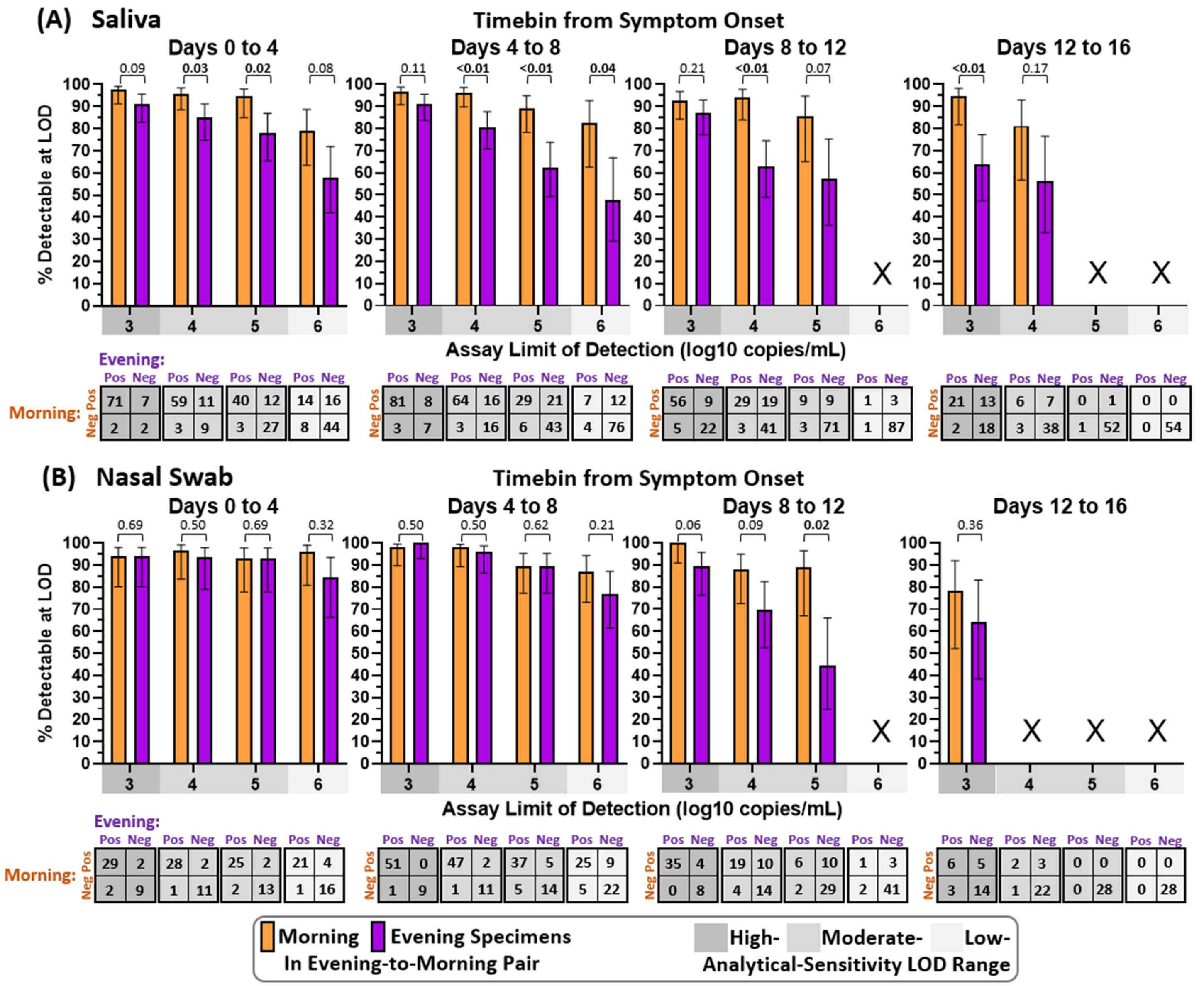

Figure S5. Morning saliva or nasal-swab specimen collection yields improved detection across infection stages and assay analytical sensitivities. For each four-day time bin relative to symptom onset, pairs of sequentially collected evening-to-morning specimens were assessed. In each pair, the viral load in each specimen was used to predict positivity if tested by an assay with a given limit of detection (LOD). Bar plots show the fraction of pairs with a positive result in either the morning or evening specimen that would be detectable if the morning specimen (orange) or evening specimen (purple) were tested at a given LOD. Error bars indicate the 95% confidence interval. Bars are not shown (X) when fewer than 10 pairs had positive results at the given LOD during the infection time bin. Among LODs and infection time bins with more than 10 positive pairs, the percent detectable for morning versus evening specimens were compared by upper-tailed McNemar Exact Test, applied to the 2x2 table shown below each comparison. Resulting P-values are shown above each comparison. Bolded values indicate significantly higher detection with morning sampling over evening sampling. Analysis was performed on **(A)** saliva specimens and **(B)** nasal swab specimens. Equivalent analysis for morning-to-evening pairs is shown in **Figure 3**.

AUTHOR CONTRIBUTIONS (listed alphabetically by last name):

Reid Akana (RA): collaborated with AVW in creating digital participant symptom surveys; assisted with data quality control/curation with JJ, NWS, NS; created current laboratory information management system (LIMS) for specimen logging and tracking. Creation of iOS application for specimen logging/tracking. Configured an SQL database for data storage. Created an Apache server and websites to view study data. Configured FTPS server to catalog PCR data. Wrote a Python package to access study data. Worked with ESS, AVW, AER to implement logic that prioritized specimen extraction order.

Jacob T. Barlow (JTB): Created initial specimen tracking database to aid in specimen logging and tracking. Maintenance of database and implementation of corrections.

Alyssa M. Carter (AMC): Received, performed QC on, and logged specimens. Performed nucleic acid extractions and RT-qPCR. Aliquoting and preparing study reagents as needed. Performed preliminary experiments to assess RNA stability in our saliva and nasal swab specimens. Summarizing daily RT-qPCR data of participant time courses to inform participant keep/drop decisions. Provided feedback on early figure drafts.

Matthew M. Cooper (MMC): Collaborated with AVW, MF, NS, YG, RFI, on study design and recruitment strategies. Co-wrote initial IRB protocol and informed consent with AVW and NS; assisted in the writing of the enrollment questionnaire; developed laboratory specimen processing workflow for saliva with AVW and AER; performed specimen processing on subset of specimens; funding acquisition; collaborated with AER to write data processing/visualization code for observing household transmission events for active study participants. Contributor to the design of the calibration curve for saliva LOD experiments. Performed specimen logging and QC.

Matthew Feaster (MF): Co-investigator; collaborated with AVW, MMC, NS, YG, RFI on study design and recruitment strategies; provided guidance and expertise on SARS-CoV-2 epidemiology and local trends.

Ying-Ying Goh (YG): Co-investigator; collaborated with AVW, MMC, NS, MF, RFI on study design and recruitment strategies; provided guidance and expertise on SARS-CoV-2 epidemiology and local trends.

Rustem F. Ismagilov (RFI): Co-investigator; collaborated with AVW, MMC, NS, MF, YYG on study design and recruitment strategies; provided leadership, technical guidance, oversight, and was responsible for obtaining funding for the study.

Jenny Ji (JJ): Researched epidemiological survey structures, performed epidemiological literature review with MMC and AVW, and co-wrote enrollment questionnaire with NS and AVW. Major contributor to curation of participant symptom data. Provided quality control of participant data with RA, NS, NWS.

Michael K. Porter (MKP): Performed specimen logging and QC, RNA extractions, RT-qPCR, data processing. Performed data acquisition and analysis for and made Figure S2 with AVW. Prepared participant specimen collection materials and helped with supplies acquisition. Assisted in literature analysis with ESS, RA, AVW. Performed data analysis and prepared Fig. 2 with AVW. Assisted in preparation of Fig. 1, 3, S2, S3 with AVW. Verified the underlying data with AVW and NS. Outlined and wrote manuscript with AVW.

Jessica A. Reyes (JAR): Lead study coordinator; collaborated with NS, AVW, NWS, and RFI on recruitment strategies, translated study materials into Spanish, co-wrote informational sheets with AVW and NS; created instructional videos for participants; enrolled and maintained study participants with NS and NWS.

Anna E. Romano (AER): Developed laboratory swab specimen processing workflow with ESS. Optimized extraction protocols working with vendor scientists. Created budgets and managed, planned, and purchased reagents and supplies; developed and validated method for RT-qPCR analysis for saliva and nasal-swab specimens with MMC, and AVW. Performed specimen logging and QC, RNA extractions, RT-qPCR; Design of saliva calibration curve experiment. Managing logistics for the expansion of the BSL-2+ lab space with ESS. Provided feedback on earlier manuscript revision and provided a few references.

Emily S. Savela (ESS): Coordinated laboratory team schedules and division of lab work. Performed initial nasal-swab workflow validation experiments with AER. Major contributor to workflow validation, methods, biosafety SOPs. Developed/implemented system for specimen archiving. Performed specimen logging and QC, RNA extractions, RT-qPCR, and data processing. Managing logistics for the expansion of the BSL-2+ lab space with AER and biohazardous waste pickups.

Natasha Shelby (NS): Study administrator; collaborated with AVW, MMC, RFI, YG, MF on initial study design and recruitment strategies; co-wrote IRB protocol and informed consent with AVW and MMC; co-wrote enrollment questionnaire with AVW and JJ; co-wrote participant informational sheets with AVW and JAR; enrolled and maintained study participants with JAR and NWS; study-data quality control, curation and archiving with RA, JJ, and NWS; reagents and supplies acquisition; assembled Table 1 with AVW; managed citations and reference library; verified the underlying data with MKP and AVW; edited the manuscript.

Noah W. Schlenker (NWS): Study coordinator; collaborated with NS, AW, JAR, and RFI on recruitment strategies; enrolled and maintained study participants with NS and JAR; study-data quality control, curation and archiving with RA, JJ, and NS.

Colten Tognazzini (CT): Coordinated the recruitment efforts at PPHD with case investigators and contact tracers; provided guidance and expertise on SARS-CoV-2 epidemiology and local trends

Alexander Viloria Winnett (AVW): Collaborated with MMC, NS, RFI, YG, MF on initial study design and recruitment strategies; co-wrote IRB protocol and informed consent with MMC and NS; co-wrote enrollment questionnaire with NS and JJ; co-wrote participant informational sheets with NS and JAR and digital survey; developed and validated methods for saliva and nasal-swab specimen collection; developed and validated methods for RT-qPCR analysis for saliva and swab specimens with AER, ESS, MMC; reagents and supplies acquisition; funding acquisition; developed laboratory specimen processing workflow with AER, ESS, and MMC; performed specimen logging and QC, nucleic acid extraction, RT-qPCR; assembled Table 1 with NS; analyzed viral load timeseries data to visualize trends (Fig. 1) with MKP; assisted MKP in the preparation of Fig. 2; analyzed viral load data to generate Fig 3 with MKP; analyzed specimen collection data for Fig S1; generated longitudinal RT-qPCR plot array for Fig S2, Fig S3 with MKP; analyzed data for Fig S4. Verified the underlying data with MKP and NS. Outlined and wrote manuscript with MKP.
